# 

**Published:** 1898-02

**Authors:** 


### CONTENTS.

ORIGINAL ARTICLES: PAGE
A Few Words Anent Wolf-Teeth. By Dr. L. A. Merillat . . . . . . 67
Thermo-cautery, and its use in Veterinary Practice. By P. O. Koto, M.D.C. . 73
Lymphoma. By J. L. Miller, V.S. . . . . . . . . . . . . 78
Feeding Corn-smut to Dairy-cows. By Clinton D. Smith . . . . . . 80
ABSTRACTS FROM FOREIGN JOURNALS:
Contagious Cerebro-spinal Meningitis of the Horse . . . . . . . . 87
Analogy of Irian Uveitis in Man, with Dry Periodic Fluxion in the Horse . . 89
Actinomycosis of the Udder . . . . . . . . . . . . 90
Monodactyle Pigs . . . . . . . . . . . . . . 91
Enzootic Gastro-enteritis from Acorns . . . . . . . . . 91
Accidental Death from Morphine . . . . . . . . . . . 91
Verminous Typhlitis . . . . . . . . . . . . . . 91
The Phonendoscope . . . . . . . . . . . . . . 92
A Long Fast . . . . . . . . . . . . . . . 92
REPORTS OF CASES:
Transverse Fracture of the Axis. By G. L. Warner, D.V.S. . . . . . 93
I. Ante-partum Vaginal Prolapse. II. Excessive Œdema of the Head. By W. H. Dalrymple, M.R.C.V.S. . . . . . . . . . . . . . 94
I. Mediastinal Abscess in a Cow Simulating Traumatic Pericarditis. II. Which Testicle is most Frequently Displaced in Cryptorchidism? By S. J. J. Harger, V.M.D. . . . . . . . . . . . . . . . . . 96
Two Cases of Lameness in ihe Region of the Femero-tibial Articulation. By W. E. A. Wyman, V.S. . . . . . . . . . . . . . . 97
Remedies Worth Trying in Eczematous Eruptions of Dogs . . . . . . . 99
EDITORIALS:
Losses in the Veterinary World—For the Best-equipped Meat-inspectors—An Important Meeting—Omaha, 1898 . . . . . . . . . . . . 100
NEW PUBLICATIONS—CONTROL WORK—LEGISLATION—GLEANINGS—HERE AND THERE . . . . . . . . . . . . . . . 103
SOCIETY PROCEEDINGS:
Western Ontario Veterinary Medical Association—Ontario Veterinary Medical College Society—Chicago Veterinary Society—Schuylkill Valley Veterinary Medical Association—Massachusetts Veterinary Association—Veterinary Medical Association of New York County—Veterinary Medical Society of the University of Pennsylvania—Keystone Veterinary Medical Association—U. S. V. M. A.—Ninth International Congress of Hygiene and Demography—Society Notes . . 116
PERSONALS—NEW INVENTIONS . . . . . . . . . . . . 128

